# Effects of Herbs and Derived Natural Products on Lipopolysaccharide-Induced Toxicity: A Literature Review

**DOI:** 10.1155/2023/7675183

**Published:** 2023-04-17

**Authors:** Majid Kianmehr, Mohammad Behdadfard, Mahdiyeh Hedayati-Moghadam, Mohammad Reza Khazdair

**Affiliations:** ^1^Esfarayen Faculty of Medical Sciences, Esfarayen, Iran; ^2^Student Research Committee, Birjand University of Medical Sciences, Birjand, Iran; ^3^Department of Physiology, School of Medicine, Jiroft University of Medical Sciences, Jiroft, Iran; ^4^Cardiovascular Diseases Research Center, Birjand University of Medical Sciences, Birjand, Iran

## Abstract

**Introduction:**

Oxidative stress (OS) during inflammation can increase inflammatory responses and damage tissue. Lipopolysaccharide (LPS) can induce oxidative stress and inflammation in several organs. Natural products have several biological activities including anti-inflammatory, antioxidant, and immunoregulatory properties. The aims of the study are to study the possible therapeutic effects of natural products on LPS inducing toxicity on the nervous system, lung, liver, and immune system.

**Methods:**

The *in vitro* and *in vivo* research articles that were published in the last 5 years were included in the current study. The keywords included “lipopolysaccharide,” “toxicity,” “natural products,” and “plant extract” were searched in different databases such as Scopus, PubMed, and Google Scholar until October 2021.

**Results:**

The results of most studies indicated that some medicinal herbs and their potent natural products can help to prevent, treat, and manage LPS-induced toxicity. Medicinal herbs and plant-derived natural products showed promising effects on managing and treating oxidative stress, inflammation, and immunomodulation by several mechanisms.

**Conclusion:**

However, these findings provide information about natural products for the prevention and treatment of LPS-induced toxicity, but the scientific validation of natural products requires more evidence on animal models to replace modern commercial medicine.

## 1. Introduction

Lipopolysaccharide (LPS) is a central component of the outer membrane in Gram-negative bacteria which causes inflammation [1]. LPS is a glycolipid in the outer membrane that provides an effective permeability barrier against deleterious molecules such as antibiotics. The LPS molecule has a tripartite structure including (i) lipid A, the hydrophobic moiety that anchors LPS to the outer membrane, (ii) core oligosaccharide, which contributes to maintaining the integrity of the outer membrane with together lipid A, and (iii) O antigen (O antigen polysaccharide), which consist of a polymer made of repeating oligosaccharide units and connected to the core in direct contact with the external milieu [2].

Systemic administration of LPS could induce disorder in several organs [3, 4]. The degeneration of the proinflammatory mediators was increased due to LPS via nuclear factor kappa-light-chain-enhancer of activated B cells (NF-*κ*B) pathways [5]. LPS also mediated immune response due to the activation of Toll-like-receptor 4 (TLR4) [6]. LPS/TLR4 signaling induces the production of the proinflammatory cytokine [5]. So, the changes in proinflammatory mediator levels in the serum are important indicators of the immune response due to LPS administration [7]. LPS also declined the density of nicotinic acetylcholine receptors of the *α*7 subtype (*α*7 nAChRs) in the brain as well as reduced nucleated cell numbers in the hippocampus and striatum in mice [8].

It was reported that LPS-treated animals induced liver and heart toxicities by activation of NF-*κ*B and toxic mediators generated by the activated macrophages (proinflammatory molecules and cytokines releases) and activation of reactive nitrogen and oxygen species (RNS and ROS) including superoxide and nitric oxide (NO) radicals [9, 10]. LPS could stimulate ROS pathways by a mediated protein-tyrosine kinase (PTK), protein kinases (PK), and mitogen-activated protein kinase (MAPK) [11].

Administration of LPS also causes lung damage through several inflammatory mechanisms [12]. Oxidative stress (OS) and inflammation are important components of lung diseases, including pulmonary fibrosis, asthma, and acute lung injury (ALI) [13]. The imbalance between cellular antioxidative capacity and ROS formation occurred in OS. It was reported that the interplay between OS and inflammation resulted in the progression of several disorders [14] which have an important function in ALI induced by LPS. Also, OS causes the recruitment of inflammatory cells including macrophages and T lymphocyte-mediated production of proinflammatory mediators such as interferon-gamma (IFN-*γ*), interleukin (IL)-1b, and IL-12, that lead to inflammation [15]. LPS signaling is highly relevant to the pathophysiology of many chronic inflammatory diseases. LPS influences a range of cell types and physiological processes such as the immune system, neural system, lung, liver, and cardiovascular system [16].

Recent studies suggest that the use of natural products as antioxidant and anti-inflammatory agents has beneficial effects in ameliorating responses to inflammatory insults of LPS-induced toxicity [17]. Then, it is logical that inhibition of inflammation and OS is an effective strategy to reduce LPS-induced tissue damage.

It has been reported that almost 7,000 active natural compounds have been derived from medicinal herbs [18]. The major advantage of herbal drugs (active natural compounds derived from herbs) is the lower side effects compared to chemically synthesized drugs [19]. Phytomedicine, due to its antioxidant, anti-inflammatory, and immunoregulatory effects, was used for the treatment of various diseases [20–22]. The effect of thymoquinone (TQ), the main component of *Nigella sativa* seeds, on LPS-induced lung injury in rats significantly improved lung pathological changes and the levels of cytokines (TGF-*β*1, INF-*γ*, PGE2, and IL-4) in bronchoalveolar lavage fluid (BALF) compared to the LPS-treated group [23]. The effects of hydroethanol extract *Zataria multiflora* on learning and memory capacity in LPS-challenged rats also remarkably reduced IL-6, malondialdehyde (MDA), and nitric oxide (NO) metabolite concentrations, but increased thiol content, superoxide dismutase (SOD), and catalase (CAT) activities [24].

The aim of this study was to review the evidence on the role of herbs and derived natural products in the treatment or reducing the adverse effects of LPS-induced toxicity on different organs.

## 2. Method

The information of this study was obtained by searching the keywords such as “lipopolysaccharide”, “natural products”, “medicinal plant”, and “toxicity” in different online databases including Google Scholar, PubMed, Science Direct, and Scopus as follows: (TITLE-ABS-KEY (lipopolysaccharide) AND TITLE-ABS-KEY (induced AND inflammation) AND TITLE-ABS-KEY (natural AND products)) in Scopus: 406 ((lipopolysaccharide [Title/Abstract]) AND (inflammation [Title/Abstract])) AND (natural products [Title/Abstract]), Pubmed: 87.

## 3. Effects of Natural Products on LPS-Induced Neuroinflammation

### 3.1. In Vitro Studies

Neuroinflammation accompanies and often precedes the development of neurodegenerative diseases including Parkinson's and Alzheimer's diseases (AD) and might be one of the pathogenic factors for neurodegeneration [25].

Pretreatment of rat brain microglia cells exposed by LPS with crocin (20 *μ*M) and crocetin (20 *μ*M) separately, markedly decreased hyperproduction of NO, tumor necrosis factor-*α* (TNF-*α*) and interleukin (IL)-1*β*. Also, crocin and crocetin (20 and 40 *μ*M) could inhibit the LPS-induced activity of NF-*κ*B [26]. Microglia activation has a critical role in the pathology of neurodegenerative disorders in considering the close correlation of excessive microglia activation with the secretion of proinflammatory mediators and neurotoxic factors [27]. A study by Liu et al. [28] showed a significant reduction of TNF*α* and NO levels in neuron-glia cells pretreated with the extract of *Tripterygium wilfordii* (a Chinese herb) due to improvement of microglia function [29]. Tripchlorolide (1.25–10 nM), extracted from *Tripterygium wilfordii*, was able to increase cell survival via inhibition of inflammatory enzymes including cyclooxygenase-2 (COX-2) and inducible nitric oxide synthase (iNOS). Tripchlorolide suppressed the production of TNF-*α*, IL-1b, prostaglandin E2 (PGE2), NO, and intracellular superoxide anion in LPS-stimulated neuro-2A cells and primary cortical neurons [30].

LPS with activation of inflammatory mechanisms in the substantia nigra (a midbrain dopaminergic nucleus) and degeneration of motor neurons are used for induction of Parkinson's disease models. Administration of myricetin (12.5, 25, and 50 micromoles (*μ*M)) as lipophenolic compounds extracted from red wine on a murine microglia cell line (BV-2) exposed to LPS reduced mRNA and the protein levels of the TNF-*α*, IL-1*β*, and IL-6 in the cells [28].

Usage of isoflavone, Biochanin A (1.25, 2.5, 5 *μ*M) mainly found in red clover (a flowering plant) for 21 days in rat primary microglia exposed to LPS decreased the levels of NO, TNF-*α*, ROS, and IL-1b in microglial culture [31].

Licochalcone A, a flavonoid isolated from licorice roots, changed neuroglia inflammatory circuits in BV-2 cells by inhibiting phosphorylation of NF-*κ*B p65 and Ras-dependent extracellular signal-regulated kinase (ERK1/2) [32].

LPS also could induce NO production via an increase of activity of iNOS and p38 mitogen-activated protein kinase (MAPK) signaling pathway. Silymarin, a milk thistle polyphenolic flavonoid, decreased levels of TNF-*α* and NO in mesencephalic LPS-stimulated mixed neuron–glia cultures. Silymarin treatment also inhibited NF-*κ*B activation and superoxide generation with any change in the p38 MAPK signaling pathway [33].

### 3.2. In Vivo Studies

The result of the Morris water maze and passive avoidance (based on negative reinforcement and used to study memory) showed that administration of a monoterpenoid phenol (carvacrol) improved memory and learning disturbances as well as brain tissue inflammation and OS in LPS-exposed rats. Intraperitoneal (IP) injection of carvacrol (25, 50, and 100 mg/kg, IP) 30 min before LPS administration significantly reduced NO, IL-6, and malondialdehyde (MDA) level and increased catalase (CAT) and superoxide dismutase (SOD) activity, as well as total thiol content in brain tissue of rats, inoculated with LPS [34]. Lee et al. (showed daily administration of carvacrol (25-100 mg/kg, IP) for 21 days in neuroinflammation induced by LPS reduced the expression of IL-1*β*, TNF-*α*, and COX-2 in the brain of rats. In addition, carvacrol decreased the expression of TLR4 mRNA and increased the mRNA expression of brain-derived neurotrophic factor (BDNF) [35].

Gypenosides (the main active components of Gynostemma species) as anti-inflammatory agents was useful for the treatment of neuropsychiatric disease, such as anxiety. Treatment with gypenosides (25-100 mg/kg), for 21 days improved behavior test results in rats with chronic inflammation induced by intracerebroventricularly (ICV) injection of LPS. Gypenosides administration increased the percentage of open arm entries and time spent in the plus maze test that commonly used behavioral assays to evaluate anxiety-related behavior in rodents. Furthermore, a decrease in brain proinflammatory mediators level including NF-*κ*B, IL-6, and IL-1*β* as well as low expression of TLR4 and BDNF mRNA in LPS- injected rats caused by gypenosides [36].

Treatment with aqueous *Clinacanthus nutans* extract (500 and 1000 mg/kg) for 14 days resulted in a significant alteration in neuroinflammation metabolite biomarkers in LPS-injected rats. Improvement in the metabolism of isoleucine, leucine, valine, and pyruvate as well as regulation of metabolic pathway glycolysis, gluconeogenesis, and TCA cycle was done by *Clinacanthus nutans* oral treatment [37]. Neuroinflammation and OS have main roles in the pathogenesis of Alzheimer's disease. LPS also due to inducing inflammation and the OS process could induce memory loss. Choi et al. reported the memory impairment induced by LPS could be ameliorated by properties of antioxidant, antiamyloidogenic, and anti-inflammatory effects of *Euphausia superba* [38] and *Nannochloropsis oceanica (N. oceanica)* [39]. Results of both studies showed administration of *Euphausia superba* (*E. superba*) oil (80 mg/kg/day), for 30 days and *N. oceanica* (50, 100 mg/kg), for 4 weeks, separately improved cognitive impairment in IP administration of LPS in mice via down expression of COX-2 and iNOS as well as decrease the level of MDA. Furthermore, *E. superba* oil and *N. oceanica* extract prevented amyloidogenesis by suppressing p50 and p65 translocation into the nuclei of brain cells. In addition, *E. superba* oil and *N. oceanica* extract inhibited amyloid protein precursor (APP) and *β*-site APP cleaving enzyme (BACE1) expression in brain cells of LPS-injected mice. The APP and BACE1 have main roles in the generation of amyloid beta, an insoluble peptide that accumulated in the brain of neurodegenerative disorders [38, 39].

Kong et al. showed the LPS induced neurotoxicity and neuroinflammation in mice hippocampus, HT22 cells, and BV-2 cells via activation of C-Jun NH2 terminal kinase (JNK), WDFY1/TLR3, and NF-*κ*B signaling cascades. Treatment with a glycoside isolated from *Forsythiae fructus* (Forsythoside B), prevented LPS function in the activation of inflammatory mechanisms [40].

*In vitro* and *in vivo* experiments showed a sesquiterpene lactone, isolated from *Centipeda minima,* 6-Oangeloylplenolin (0.5−4 *μ*M), has anti-inflammatory and anti-oxidative properties in cell line and mice associated with LPS induced neuroinflammation and stimulated secretion of TNF-*α*, IL-1*β*, NO, and PGE2 in BV2 and primary microglial cells. This study also showed 6-Oangeloylplenolin could improve synaptic connections and function of neurons and neuroglial in the central nervous system due to attenuate of LPS-induced NF-*κ*B activation. Pretreatment of mice with 6-Oangeloylplenolin (5-20 mg/kg, IP) for 7 days before LPS injection prevented cytokine secretion via inhibition of the NF-*κ*B signaling pathway and downregulation of inflammatory enzymes in brain cells [41]. LPS with activation of inflammatory mechanisms in substantia nigra and degeneration of motor neurons was used for induction of Parkinson models. Treatment of LPS-induced Parkinson model rat with myricetin, ameliorated rat's motor dysfunction. In addition, the downregulation of microglial inflammatory mediator expression due to MAPK and NF-*κ*B pathways suppressed by myricetin caused to compensate for LPS-induced reduction in dopaminergic neurons number [28].

The usage of biochanin A (an O-methylated isoflavone) for 21 days improved the results of behavioral tests in Parkinson's disease model rats induced by LPS. Biochanin A could protect dopaminergic neurons via inhibition of cytokine secretion such as TNF-*α*, IL-1b, and IL-6 and suppress of ERK, JNK, and p38 phosphorylation and block MAPK signaling pathway in Parkinson's disease rats. Results of *in vitro* study also showed the levels of NO, TNF-*α*, ROS, and IL-1b decreased in supernatants of microglial culture by biochanin A treatment [31]. LPS with an increase of microglial activity, a decrease in dopamine uptake, and tyrosine hydroxylase activity could induce Parkinson's disease. Treatment with a phenol chalconoid extracted from the roots of Glycyrrhiza species, Licochalcone A (1.25, 2.5, and 5 mg/kg, IP), attenuated behavioral deficits in female Wistar rats that have been injected LPS to substantia nigra [32].

**Figure 1:**
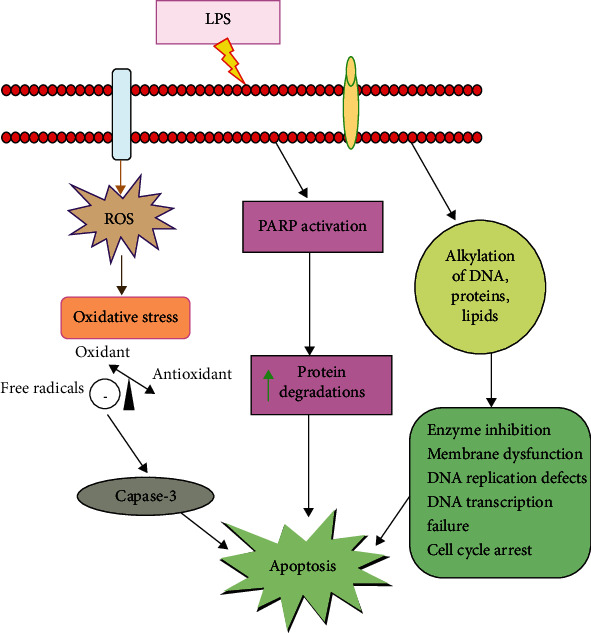
Lipopolysaccharide- (LPS-) induced neurotoxicity. Reactive oxygen species (ROS). Poly (ADP-ribose) polymerase (PARP).

An animal study showed *Polygala tenuifolia* which has anti-inflammatory and antioxidant properties could protect dopaminergic neurons against the progression of Parkinson's disease. Usage of tenuigenin (300 mg/kg, 14 weeks), an active component of *Polygala tenuifolia*, prevented LPS-induced microglia activity and increased dopamine content in the striatum of LPS-injected rats. Furthermore, tenuigenin was able to decrease the levels of TNF-*α* and IL-1b in the substantia nigra pars [42]. Upregulation of TLR4 is associated with initiating the immune response in the microglia resulting in neuronal death and apoptosis. LPS could induce expression of TLR4 in the microglial following an ischemia stroke. Induction of TLR4 caused increased cytokines production via increased activity of proinflammatory pathway mediators such as NF-*κ*B, MyD88, ERK1/2, JNK, p38, and MAPK. Treatment with active component extracted from Flos *Carthami tinctorii* (Hydroxysafflor yellow A), by downregulation TLR4 pathway-mediated signaling components and also upregulation of BDNF, alleviated LPS-induced neuroinflammation in microglia as well as degeneration in neurons [43]. The results of various *in vitro* and *in vivo* studies indicated that some medicinal herbs and plant ingredients showed neuroprotective effects by anti-inflammatory and antioxidant properties in LPS-induced neurotoxicity. The effects of medicinal plants and plant ingredients on LPS-induced neuroinflammation were summarized in Table 1 and Figure 1.

## 4. Effects of Natural Products on LPS-Induced Lung Injury

### 4.1. In Vitro Studies

Procyanidin B2 (PCB2) is a multifunctional natural dietary phytochemical at doses (5, 10, and 20 mM) that inhibited activation of NF-*κ*B and reduced expression and release of TNF*α* and IL-1*β* in LPS-treated human alveolar epithelial cells (AECs). PCB2 also prevented LPS-induced NLR family pyrin domain containing 3 (NLRP3) inflammasome activation and reduced ROS generation in human vascular endothelial cells. PCB2 enhanced Bcl-2 expression while inhibiting LPS-induced Bax and active caspase-3 production. The researchers found that in LPS-treated human AECs, PCB2 inhibited the activation of the NF-*κ*B and NLRP3 inflammasomes, suggesting that it may have therapeutic potential for acute lung injury (ALI) [44].

Lately, the effect of Lonicerin (LCR) has been examined on the nontumorigenic human lung epithelial cell line (BEAS-2B). LCR (0 to 160 *μ*M) significantly decreased the production of TNF-*α*, IL1, and IL6. LCR pretreatment significantly reduced the number of p-NF-*κ*B expressing cells compared to LPS-treated cells. According to studies, LPS elevated the expression of caspase-3 and poly (ADP-ribose) polymerase (PARP), which was considerably controlled by LCR pretreatment. In BEAS-2B cells, LCR also reversed the impact of LPS on the expression of Bax and B-cell lymphoma 2 (Bcl-2). Pretreatment with LCR might prevent LPS from activating the apoptotic pathway [45].

### 4.2. In Vivo Studies

Methanol extract of *Spiraea prunifolia* (SP) leaves which were examined on NCI-H292 cells in a mouse model (0, 10, 25, 50, and 100 *μ*g/mL) decreased IL-1*β*, IL-6, and the levels of TNF-*α* in the bronchoalveolar lavage fluid (BALF). In LPS-induced ALI mice, SP dramatically reduced the phosphorylation of MAPKs and NF*κ*B. SP therapy increased Nrf2 elevated antioxidant enzymes and reduced ROS-mediated OS in LPS-induced ALI. Treatment with SP showed a considerable reduction in ROS generation and a large increase in glutathione (GSH) and 2,2-diphenylpicrylhydrazyl (DPPH) radical scavenging activities. SP decreased lipid peroxidation by activating nuclear factor erythroid 2–related factor 2 (Nrf2) and upregulating antioxidant enzymes such as heme oxygenase 1 (HO-1) and NAD(P)H dehydrogenase (quinone 1) (NQO1). SP's capacity to stimulate Nrf2 activation and decrease the phosphorylation of MAPKs and NF-*κ*B was associated with the ability to suppress airway inflammation [46].

According to Mo et al., S-allylmercaptocysteine (SAMC) can reduce LPS-induced ALI in mice by reduction of inflammation via NF-*κ*B and Nrf2 pathways. According to their findings, TNF-*α*, IL-1*β*, and IL-6 levels were reduced after treatment with SAMC (10, 30, and 60 mg/kg, IG). Also, treatment with SAMC enhanced the levels of SOD and GSH while inhibiting the generation of MDA. SAMC treatment considerably reduced inflammatory cell infiltration and decreased the notable elevation of metropolitan planning organization (MPO), COX2, and iNOS caused by LPS. The administration of SAMC reduced the infiltration of LPS-induced inflammatory cells such as macrophages and neutrophils, revealing that SAMC might effectively cure LPS-induced ALI [47].

Robustaflavone-4′-dimethyl ether (RDE) from *Selaginella uncinata* suppressed FLT3 (a member of the type III receptor tyrosine kinase) activation by interacting with a generation of inflammatory cytokines and preventing neutrophil activation in ALI mice. RDE (50, 100, and 200 mg/kg, intragastric (IG)) reduced neutrophil-endothelial cell contacts and even neutrophil infiltration in lung tissues and serum levels of IL-6 and TNF-*α* and adhesion molecules, showing that RDE suppressed LPS-induced neutrophil stimulation. MPO activation was reduced by RDE administration in a dose-dependent manner. These findings showed that RDE could suppress FLT3 expression, then inhibit the LPS-induced AKT (Protein kinase B**)** and MAPK pathways. In a dose-dependent way, RDE may reverse the elevated levels of P-selectin, IL-6, TNF-*α*, and intercellular adhesion molecule 1 (ICAM-1) caused by LPS stimulation and ameliorate lung pathologic characteristics [48].

Wang et al. showed that LPS significantly activated the COX2/NLRP3/NF-*κ*B pathway in lung tissues. Per os (PO) or oral administration of eriodictyol (20, 40, and 80 mg/kg, PO) in ALI induced by LPS in mice significantly suppressed the COX2/NLRP3/NF-*κ*B pathway. eriodictyol also inhibited the release of PGE2, TNF-*α*, IL-6, and IL-1 into BALF, which were generated by LPS. The levels of MPO and MDA, total cells, and neutrophils were significantly reduced by eriodictyol. Moreover, the histopathologic lesions decreased in the eriodictyol groups. Meanwhile, in the LPS group, SOD activity was observed to be decreased, but eriodictyol considerably enhanced SOD levels. eriodictyol also improved lung pathological abnormalities and reduced the W/D ratio. The effect of eriodictyol on ALI was proven to be mediated through decreasing the inflammatory response. The suppression of COX2/NLRP3-mediated NF-*κ*B signaling by eriodictyol causes a reduction in inflammatory cytokine production [49].

The anti-inflammatory effect of a standardized extract of *Euphorbia cuneata* (EC) against LPS-induced ALI in mice was evaluated. Total and differential cell counts were decreased by EC. Treatment with EC (25 and 50 mg/kg, PO) significantly increased CAT, SOD, and GSH while significantly decreasing MDA and 4-hydroxynonena (4-HNE) compared with the LPS-treated group. The capacity of EC to decrease NF-*κ*B and COX activation revealed its anti-inflammatory properties. In comparison to the untreated LPS group, EC administration reduced the W/D ratio, total protein, and LDH activity. EC was also found to have antioxidant properties as it reduced lipid peroxidation. EC also reduced infiltration of inflammatory cell, lung edema, and histological changes, in addition to suppressing inflammatory mediator production (TNF-*α*, IL-8, IL-4). Overall, the study showed that EC has a protective effect against LPS-induced ALI, which might relate to its t anti-inflammatory and antioxidant properties [50]. The ethanol extract of *Trichilia martiana* C. DC. (TMEE) in LPS-induced ALI male C57BL/6 N mice in the LPS-treated group elevated TNF-*α* level in the LPS group, but TMEE administration (5, 10, 20, and 40 g/ml, PO) significantly decreased this, suggesting that TMEE modulates LPS-induced macrophage influx and TNF-*α* expression. Following TMEE therapy, the amount of I*κ*B activation was dramatically reduced. TMEE also dramatically decreased the upregulation of ERK and JNK in LPS-induced ALI animals. The findings showed that TMEE-induced HO-1 production was linked to a reduction in the inflammatory response caused by LPS. The downregulation of MAPKs (particularly ERK and JNK)/NF-B signaling pathways and the activation of HO-1 appear to be linked to TMEE's actions [51].

Treatment of induced ALI mice with nanoemulsions containing pequi oil (pequi-NE) (20 mg/kg, PO) decreased the levels of IL-1, TNF-*α*, IL-6, monocyte chemoattractant protein-1 (MCP-1), and keratinocyte-derived chemokine (KC). The migration of leukocytes and neutrophils into the lungs is reduced by pequi-NE. Treatment with pequi-NE lowered MPO levels and recovered activation of catalase to saline control level. MDA levels dramatically reduced after treatment with pequi-NE. The results suggested that using nanoemulsions containing pequi oil to treat mice with ALI/ARDS (acute respiratory distress syndrome) caused by LPS has anti-inflammatory and antioxidant properties. The findings imply that pequi-NE might be used to treat pulmonary inflammatory diseases as an alternative therapeutic method [52].

The ethanolic extract of *Glycyrrhiza glabra* (*G. glabra*) (200 and 300 mg/kg, PO) significantly reduced total cell count and inflammatory cell migration, reduced the W/D weight ratio, reduced the total protein content, reduced the expression of TNF-*α*, IL-1, and IL-6, improved SOD activity, and significantly protected the lung injury in LPS-induced ALI female mice. *G. glabra* also reduced neutrophil infiltration in the lungs of ALI mice [53].

The potential of *Thalictrum minus* L. (TML) protecting mice against LPS-induced ALI in C57 male mice was shown. The findings indicated that TML (10, 20, and 40 mg/kg, PO) decreased the lung W/D weight ratio and reduced inflammatory cell tissue infiltration, which revealed that TML could help treat pulmonary edema. TML significantly reduced LPS-induced inflammatory cytokines such as TNF-*α* and IL-1, decreased NO, and elevated SOD, and effectively alleviated LPS-induced increases in total protein, leukocytes, and macrophages, according to the researchers. Bax and LC3II expressions were notably decreased, whereas Bcl-2 protein levels were elevated. TML protected against LPS-induced ALI through regulating AMPK-Nrf2/KEAP (AMP-activated protein kinase) and MAPKs p38-NLRP3/caspase1 signaling pathways and suppressing apoptosis and autophagy [54].

The results of a study on male BALB/c albino mice showed that tovophyllin A (TA) protects mice from LPS-induced ALI, which may be due to anti-inflammatory and antioxidant characteristics. Differential and total inflammatory cell counts, protein content, LDH (lactate dehydrogenase) activity, and the lung W/D ratio were all reduced by TA (50 or 100 mg/kg, PO) in LPS-induced mice. TNF-*α*, IL-1, and IL-6 levels lowered by TA. Moreover, TA improved lung lesions and enhanced antioxidant levels such as GSH and SOD [55].

According to earlier researches on male BALB/c mice, peiminine (1, 3, and 5 mg/kg, IP) reduced LPS-induced inflammatory cell aggregation and decreased MPO activity. Peiminine has been shown to reduce the generation of inflammatory cells, macrophages, and neutrophils. Additionally, peiminine therapy reduced histopathological alterations and TNF-*α*, IL-1, and IL-6 expression. During peiminine therapy, the lung W/D ratio was clearly lower. The findings showed that peiminine decreases inflammatory response injury to the body and even the risk of pulmonary edema. Peiminine might reduce ALI caused by LPS by blocking the PI3K/AKT/NF-*κ*B signaling pathway [56].

Administration of *Nigella sativa* (NS) extract on LPS-exposed male Wistar rats decreased IFN-*γ*, PGE2, and transforming growth factor beta-1 (TGF-*β*1) but increased IL-4 levels. Treatment with NS extract (100-400 mg/kg, IP) inhibits the synthesis of IL-6, TNF-*α*, and NO. In rats exposed to LPS, NS therapy enhanced the total WBC count (eosinophils, neutrophils, basophils, monocytes). Administration of NS decreased the generation of MDA and increased thiol level and also serum SOD and CAT activity. NS therapy also improved pathogenic abnormalities in the lungs [57].

Administration of narciclasine (2 mg/kg, IP) in the LPS-induced rats protected lung injury via interdiction effect on apoptosis, excessive inflammation, and OS. Narciclasine treatment also attenuated pathological injury and pulmonary edema and suppressed the secretion of IL-6, IL-1*β*, TNF-*α*, and MCP-1 as well as reduced the expression of intercellular adhesion molecule-1 (ICAM-1) and vascular cell adhesion molecule 1 (VCAM-1) in neonatal ALI rats. Besides that, narciclasine inhibited nuclear translocation of NF-*κ*B and activation of the TLR4/NF-*κ*B/Cox2 signaling pathway. Overall, narciclasine protected against lung damage by inhibiting excessive inflammation, OS, and apoptosis [58]. Chrysin (CHR) (3 mg/kg, IT) may have therapeutic potential for relieving LPS-induced ALI in mice by blocking the Inositol-requiring enzyme 1/thioredoxin interaction protein/NLRP3 (IRE1/TXNIP/NLRP3) pathway and thus decreasing inflammatory cytokine release. CHR pretreatment reduced MPO synthesis and levels of IL-1*β*, IL-6, and TNF-*α*, which reduced inflammation. Furthermore, CHR increased antioxidant capacity by enhancing SOD and glutathione peroxidase activity (GSH-Px). CHR also decreased lung edema and vascular leakage as well as the expression of TXNIP, NLRP3, and cleaved caspase-1 [59].

Zhang et al. revealed that ergosterone (15 and 30 mg/kg, PO) reduced the damage caused by the LPS model in ALI model mice by inhibiting the NLRP3 signaling pathway. The findings showed that ergosterone pretreatment reduced the W/D ratio, IL-1, IL-6, TNF-*α*, NO, and MDA levels in lung tissues while also notably increasing SOD levels. Furthermore, ergosterone reduced the activation of NLRP3 caused by LPS. The findings suggest that ergosterone protects against ALI caused by LPS, and it may suppress the NLRP3 signaling pathway's activation. Ergosterone pretreatment not only decreased OS but also improved pulmonary impairment and reduced lung edema [60]. Ferulic acid (FA) (25-100 mg/kg, IG) in LPS-treated lungs in mice significantly reduced the production of TNF-*α*, IL-1, and IL-6. Treatment with FA or dexamethasone (DEX) substantially reduced the LPS-induced lung W/D weight ratio and histo-pathological abnormalities. The invasion of total neutrophils and macrophages in the BALF of mice damaged by LPS was reduced by FA. The study showed that FA therapy could significantly reduce cell infiltration and protect against ALI caused by LPS. FA decreased pulmonary edema, activation of MPO, and monocyte chemoattractant protein-1 (MCP1**)** level in LPS-injured mice. The mechanisms of FA's protection against LPS-induced ALI were related to the reduction of inflammatory response via the inhibition of the TLR4/NF-*κ*B signaling pathway [61].

Fucoxanthin's anti-inflammatory properties have recently been expanded in mice. Through activating the Nrf2-mediated pathway, fucoxanthin (10 mg/kg, IV) notably reduced inflammatory responses in LPS-induced ALI mice. LPS induced upregulation of IL-6, IL-10, iNOS, COX-2, TNF-*α*, and IL-1*β* that was reduced by fucoxanthin via the AMPK/NF-*κ*B signaling pathway. TLR4/MyD88/NF-*κ*B signaling was inhibited by fucoxanthin, which binds directly to TLR4. Based on discoveries, fucoxanthin can considerably suppress LPS-induced inflammatory mediators [62].

Under *in vivo* condition, LCR pretreatment reduced the production of IL-6, IL-1*β*, and TNF-*α*, by controlling the activation of TLR4, myeloid differentiation factor 2 (MD2), and p-NF-*κ*B. Pretreatment with LCR (10, 20, and 30 mg/kg, IP) significantly reduced stimulated caspase-3 and PARP cleavage in the lungs of mice. Administration of LCR improved histologic changes and reduced inflammatory cell infiltration. As a result, LCR could be a new promising treatment option for ALI based on the anti-inflammatory and antiapoptotic effects [45].

Dehydrodieugenol B can reduce lung edema, inflammatory cells, IL-6 and IL-1*β* levels, inflammatory cell infiltration, iNOS, an inhibitor of metalloprotease-1 (TIMP-1), matrix metalloproteinase-9 (MMP-9), and collagen content and expression. Treatments decreased the number of total inflammatory cells, according to the findings. Dehydrodieugenol B (20-60 mg/kg/weight) prevented pulmonary remodeling in an animal model of ALI by reducing inflammatory cells, lung edema, IL-6 and IL-1*β* levels, iNOS, and collagen content and expression. Dehydrodieugenol B showed anti-inflammatory and antioxidant properties by reducing OS and inhibiting JNK [63]. It has been reported that three kinds of bioactive components in *Sarcandra glabra* (rosmarinic acid+isofraxidin+chlorogenic acid) in different doses (5 to 50 mg/kg) reduced LPS-induced phosphorylated NF-*κ*B protein expression. Also, pretreatment with three components reduced MPO but increased SOD activation and HO-1 expression. Three components inhibited the production ofIL-1*β*, IL6, and TNF-*α* and reduced the production of iNOS and COX-2 proteins. They also reduced inflammatory response by blocking macrophage activation, resulting in lower IL-6, NO, and TNF-*α* secretion [64].

The effects of Salviplenoid A (SA) in the LPS-induced ALI mice model blocked expression of activated Nrf2 and nuclear factor of kappa light polypeptide gene enhancer in B-cells inhibitor, alpha (I*κ*B-*α*) phosphorylation in a dose-dependent manner. The amount of COX-2 protein in LPS-induced ALI animals was significantly elevated, which was potently reduced by administration of SA (10-40 mg/kg, IP). In the presence of SA, the levels of HO-1 mRNA increased significantly as compared to the untreated group [65].

Treatment with thymol (20-80 mg/kg, IP) 1 h after administration of LPS reduced alveolar wall thickness and lung edema, and increased the level of infiltrated inflammatory cells in mice. Thymol also inhibited the production of IL-6, IL-1*β,* and TNF-*α*, in the BALF of mice. In addition, thymol reduced MDA content and MPO activity in the lung tissue. Thymol also significantly inhibited LPS-induced NF-*κ*B activation [66]. Pretreatment with carvacrol (20-80 mg/kg) decreased the lung wet/dry weight ratio in LPS-induced ALI in mice and significantly decreased total and different WBC similar to dexamethasone (5 mg/kg). Carvacrol also reduced inflammatory infiltration, focal area of fibrosis, and production of IL-1*β*, IL-6, and TNF-*α* in the BALF of mice [67].

The effect of hordenine (Hor) (10 mg/kg) on LPS-induced ALI inhibited the levels of IL-1*β*, IL-6, and TNF-*α* in the BALF and IL-1*β*, IL-6, iNOS, Cox2, and MPO mRNA expression levels in the lung tissues of ALI mouse model. Pretreatment with Hor significantly decreased the increased COX-2, iNOS, IL-6, and TNF-*α* levels and promoted the expression of arginase one (Arg-1), chitinase-3-like-3, and mannose receptor (CD206). Hor also reduced ALI by inhibiting the phosphorylation of phosphorylated protein kinase (AKT), NF-*κ*B, and mitogen-activated protein kinase (MAPK), [68].

**Figure 2:**
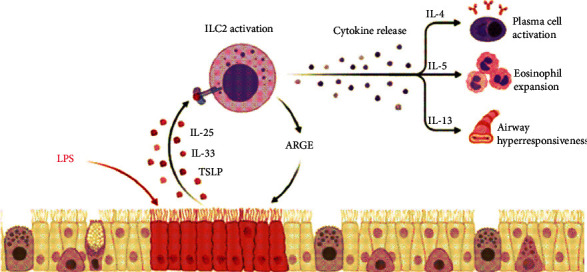
Lipopolysaccharide- (LPS-) induced lung inflammation. Thymic stromal lymphopoietin (TSLP). Innate lymphoid cells (ILC2). Amphiregulin (AREG).

The effects of munronoid I, which is extracted and purified from *Munronia sinica* on LPS-induced inflammation in mice showed administration of munronoid I (10 mg/kg, IV) significantly inhibited LPS-induced infiltration of inflammatory cells, scored of lung tissue damage, and also the production of IL-1*β* and IL-6 in BALF of ALI mice [69]. The effects of medicinal herbs and natural products on LPS-Induced lung inflammation are shown in Table 2 and Figure 2.

## 5. Effects of Natural Products on LPS-Induced Hepatotoxicity

### 5.1. In Vitro Studies

The findings of the RT-qPCR and ELISA tests demonstrated that Mangiferin (MF), a glucosylxanthone remarkably reduced LPS-induced mRNA and protein level of TNF-*α*. Additionally, reporter gene analysis revealed that MF dramatically reduced LPS-enhanced NF-*κ*B and activator protein-1 (AP-1) activity. The expression of LPS-induced TLR4 in Kupffer cells (KCs) was also downregulated by MF, according to flow cytometry data. Furthermore, the findings suggested that eme oxygenase-1 (HO-1) may downregulate the TLR4 signaling pathway, therefore regulating the anti-inflammatory action of MF in KCs [70]). The findings show that apigenin (2.5, 5, 10, and 20 *μ*M) can protect against D-galactosamine (D-GalN)/LPS-treated hepatocellular damage via increasing translocation of Nrf2 nucleus, which increases the protein expression of peroxisome proliferator–activated receptor *γ* (PPAR), CAT, and SOD amounts, and so inhibits the inflammatory reaction. The MDA content was reduced in the apigenin-treated groups. The I*κ*B-*α* and PPAR proteins expression was enhanced in the hepatocytes after treatment with apigenin. Apigenin therapy may similarly reduce the amount of TNF-*α*. To summarize, the current findings show that apigenin protects against D-GalN/LPS-enhanced liver damage [71].

In LPS-induced HepG2 cells, researchers discovered that limonin (10, 25, and 50 *μΜ*) increased cell survival *in vitro*. Hepatotoxicity induced via LPS was reduced by limonin. On the other hand, limonin suppressed the production of ROS in cells caused by LPS. The results demonstrated that LPS increased NOD-like receptor protein 3 (NLRP3) protein expression in HepG2 cells but limonin administration significantly reduced. In the presence of LPS, limonin reduced the proportion of cas-1, as well as IL-1*β*, in HepG2 cells. As a result of their evidence, limonin appears to have the potential to be a future medicine to treat liver damage [72].

### 5.2. In Vivo Studies

LPS-induced liver damage in mice was reduced by pretreatment with an ethanol extract of *Illicium henryi* (EEIH). EEIH (1.25-5.0 mg/kg, IP) dramatically reduced expression of IL-1*β*, IL-6, TNF-*α*, and COX-2 in LPS-induced ALI, through downregulating of TLR4 mRNA expression and suppressing NF-*κ*B phosphorylation. Additionally, EEIH significantly lowered the rate of nitrosative stress and liver oxidative in LPS-treated mice by lowering NO and iNOS values, upregulating Nrf2, and increasing GSH and SOD values. The blood values of alanine transaminase (ALT) and aspartate aminotransferase (AST) and MPO activity in the liver in LPS-induced mice were significantly reduced after pretreatment with EEIH. The findings showed that EEIH defends mice against ALI by decreasing the TLR4/NF-*κ*B signaling pathways and lowering the inflammatory reaction [73].

The effects of kaempferol on the ALF-induced mouse were investigated. The terminal deoxynucleotidyl transferase dUTP nick end labeling (TUNEL) assay revealed that in D-GalN/LPS-induced liver damage, the quantity of hepatocyte apoptosis had dramatically increased but kaempferol pretreatment reduced them. In the ALF-induced mouse model, kaempferol (2.5-40 mg/kg, IV) pretreatment decreased the mRNA and protein rates of C/EBP-homologous protein (CHOP), a modulator for reticulum stress- (ER stress-) induced apoptosis, according to RT-PCR and western blotting studies. Finally, kaempferol protects the mouse from ALF by inhibiting apoptotic hepatocytes through modulating the CHOP-glucose-regulated/binding immunoglobulin protein 78- (Grp78-) ER stress pathways. As a result, kaempferol may be effective in the treatment of ALF [74].

Chicoric acid (CA) was studied in the context of acute liver damage caused by LPS and d-GalN. According to the findings, CA (50 mg/kg) lowered aspartate aminotransferase (AST), alanine aminotransferase (ALT), and ROS in serum and reduced mortality caused by LPS/d-GalN. The amount of GSH in the liver elevated after treatment with CA. To reduce inflammation, CA may inhibit mitogen-activated protein kinases (MAPKs) and NF-*κ*B. Meanwhile, the findings showed that CA activated the Nrf2 pathway by raising AMP-activated protein kinase (AMPK) levels. The CA suppressed the NLRP3 and ASC, so the rate of cas-1 was reduced in the treatment group. CA therapy significantly enhanced the protein levels of autophagy genes [75].

It was reported that myricetin (Myr) (25-100 mg/kg, IP) protected fulminant hepatitis (FH) in mice against LPS/D-GalN-induced by reducing mortality, lowering serum AST and ALT levels, and reducing histological alterations, inflammation (IL-1*β*, IL-6, and TNF-*α*), OS, and liver apoptosis. Furthermore, in mice with LPS/D-GalN-induced FH, Myr could effectively moderate multiple signaling pathways, not only regulation of P53 protein and caspase-3/9, NF-*κ*B activation, and MAPK and inhibition of TLR4 but also a raise in Nrf2 and HO-1 expression, ACC, and AMPK phosphorylation. Administration of Myr could suppress NLRP3 inflammasome in mouse-induced FH [76].

In LPS-treated mice, limonin (50 and 100 mg/kg, PO) decreased serum AST and ALT activity and LDH generation while increasing hepatic GSH levels and suppressing IL-1*β* maturation. In addition, histological analysis of the liver demonstrated that limonin prevents liver failure induced by LPS. They also found that limonin reduced LPS-induced hepatotoxicity by suppressing pyroptosis through the gasdermin D/NLRP3 pathways. In conclusion, this research identified the method by which limonin reduced LPS-induced liver injury and showed that limonin might be a good candidate treatment for hepatotoxicity. [72] In LPS/D-GalN-induced mice, administration of mangiferin (MF) (30-150 mg/kg, PO) increased survival and lowered serum ALT and AST activities. MF inhibited LPS-induced TNF-*α* generation by inhibiting the NF-*κ*B/TLR4 pathways. MF therapy dramatically increased the expression and activation of HO-1 in hepatic tissues. The experiment found a possible preventive drug for LPS/D-GalN-enhanced acute liver damage [70].

Sae-tan et al. discovered that treatment with MSWE (150 mg/kg, PO) reduced the expression of iNOS in LPS-enhanced ALI mice *in vivo* studies. Expression of inflammatory cytokines such as IL1b and infiltration of macrophages had similarly reduced in the livers. TLR4 mRNA expression was shown to be considerably reduced in MSWE-treated group mice compared to the LPS group. Pretreatment with MSWE caused reduced levels of Emr1. This article showed that MSWE might be useful in the treatment of a variety of inflammatory illnesses [77]. Treatment with diosgenin (50 mg/kg, PO) might significantly decrease AST, alkaline phosphatase (ALP), and ALT concentrations in the blood. In addition, when compared to the LPS/D-Gal-treated group, hepatic rates of IL-6, IL-1*β*, MDA, ROS, NF-*κ*B, TNF-*α*, and TRL4 dramatically reduced, while the Nrf2 rate and activity of SOD improved. Furthermore, after diosgenin delivery, MPO activity, a sign of neutrophil infiltration, is reduced. In conclusion, diosgenin reduced inflammatory responses, OS, and liver damage indices, and it is considered a treatment for liver damage [78].

The NLRP3 inflammasome and the NLR family have recently been found to reduce the degree of LPS/GaIN-enhanced liver injury in mice. Shiitake mushroom-derived ELNs (S-ELNs) (1 × 10^10^/g, IP) showed to suppress activation of NLRP3 and inhibit inflammasome generation in primary macrophages. After treatment with S-ELN, the increased blood AST and ALT rates caused by GalN/LPS reduced. S-ELNs also reduced IL-18 and -6 secretion, as well as gene of IL-1*β* production. In summary, S-ELN treated mice indicated to prevent ALI caused by GalN/LPS. As a result, S-ELNs, which have been discovered as strong NLRP3 inflammasome inhibitors, demonstrated a potential effect on ALI [79].

Administration of S-allyl cysteine (SAC) (25 and 100 mg/kg, PO) in the DGal/LPS induced mice group reduced AST, ALT, and ALP and partially reduced ROS and MDA, as well as inflammation-related biomarkers such as ferric reducing antioxidant power (FRAP), MDA, liver ROS, cas-1, COX-2, TLR4, NF-*κ*B, TNF-*α*, NLRP3, IL-6 and -1*β*, and MPO function. Furthermore, SAC was able to improve apoptotic indicators such as DNA fragmentation and cas-3. In conclusion, SAC protects the liver against DGal/LPS by reducing the inflammatory response, OS, apoptotic cells, and infiltration of neutrophils, which is mainly due to the inhibition of NLRP3/NFB/TLR4 signaling [80]. Pretreatment with *Pulicaria petiolaris* (PP) (50 and 100 mg/kg, PO) resulted in a considerable decrease in LDH, AST, ALP, ALT, and CK-MB. It was discovered that pretreatment with PP considerably decreased the increased MDA concentration. Administration with PP significantly raised GSH levels and activities of SOD. Pretreatment with PP reduced NF-*κ*B p65 activity. The amounts of IL-6 and TNF-*α* dramatically reduced after pretreatment with PP. These findings implied that PP reduces LPS-enhanced oxidative injury to the liver by acting as an anti-inflammatory and antioxidative [81].

Apocynin pretreatment (300 mg/kg, IP) reduced the increase of ROS, thiobarbituric acid reactive substances (TBARS) compounds, and protein carbonyl content caused by D-Gal/LPS. Further, pretreatment with apocynin decreased the number of TUNEL-positive cells, decreased histological abnormalities, suppressed the generation of TNF-*α*, prevented the activation of the caspase downstream, and decreased mortality caused by D-Gal/LPS. Remarkably, therapy with apocynin reduced D-Gal/LPS-induced OS, apoptosis of hepatic cells, and liver damage while boosting survival. Gal/LPS-treated activation of the late-stage proapoptotic AMPK/JNK pathway was prevented by apocynin administration. These findings imply that NOX-derived ROS may be a late-stage deleterious component in D-Gal/LPS-enhanced ALI through activating the proapoptotic JNK/AMPK pathway. Apocynin may be effective in the pharmacological treatment of ALI caused by inflammation [82]. *Beta vulgaris* ethanol extract (BVEE) effectively reduced blood AST, ALT, and gamma-glutamyl transpeptidase (*γ*-GTP) levels in a rat hepatotoxicity model caused by LPS and alcohol. The elevated mRNA levels of cytochrome P450 2E1 (CYP2E1), TUNEL MAPK1, 3, *α*-Smooth muscle actin (*α*-SMA), and NF-*κ*B in the BVEE (200 and 400 mg/kg, PO) treated group had dramatically reduced. These findings suggested that BVEE might protect against hepatotoxicity by modifying several indications of liver damage caused by LPS or alcohol [83].

Curcumin (CUR) administration (200 mg/kg) resulted in a decrease in LDH, AST, and ALT activities. Furthermore, LPS/DCL caused a considerable reduction in liver GSH level and SOD activity, which was considerably restored in rats supplemented with CUR. MDA and NO levels were dramatically lowered in the livers of CUR-treated rats. CUR also decreased serum CRP, liver IL-6, TNF-*α*, NF-*κ*B, TLR4, JNK, and p38 expression while upregulating HO1. Finally, CUR protected rats against LPS/DCL-induced liver damage by reducing inflammation, TLR4 signaling, and OS while increasing HO-1. As a result, in LPS/DCL-induced rats, CUR inhibited the development and severity of liver damage while also improving liver function [84].

Administration of apilarnil (API) (0.2, 0.4, and 0.8 g/kg, PO) dramatically reduced tissue damage in LPS-induced groups. The findings showed that the API treatment group had decreased in the number of TUNEL-positive cells but the LPS-induced group had risen. The LPS-treated group raised xanthine oxidase (XOD), transcobalamin 1 (TCN1), and MDA rates while reducing CAT and SOD rates; however, API treatment inversed these effects. The LPS-induced group showed elevating in NF-*κ*B, TLR4, high mobility group box 1 (HMGB1), iNOS, IL-1*β*, IL-6, and TNF-*α* expression; however, API treatment mitigated this increment. Finally, apilarnil is considered to protect rats from LPS-induced liver injury by blocking the NF-*κ*B/TLR4/HMGB-1 pathways [85].

Administration of heptamethoxy flavone (HMF) (500 mg/kg) reduced serum ALT, ALP, AST, and MDA levels in ALI-induced rats. The antioxidant enzyme values were considerably higher in the HMF group. Furthermore, administration with HMF caused decreased rates of cas-3 and -9, Bax, Bcl-2, TNF-a, IL-b, and NF-jB in the HMF-treated rats. These findings significantly suggest that HMF might be useful for the development of a hepatoprotective medication to treat ALI [86]. Researchers found that esculetin (40 mg/kg, PO) reduced OS and myeloperoxidase activity, amount of AST, ALP, and ALT, and hepatic levels of IL-1*β*, IL-6, TNF-*α*, NF-*κ*B, and TLR4. Furthermore, esculetin reduced hepatic tissue damage after an LPS/D-Gal exposure. Esculetin has hepatoprotective properties against ALF and can prevent liver damage by reducing oxidative load, neutrophil infiltration, and inflammation [87].

According to the findings, *Platycodon grandiflorus* polysaccharides (PGPSt) (100 and 200 mg/kg, IG) dramatically lowered SOD, ALT, and AST activities. TNF-*α*, IL-1*β*, IL-6, and MDA amounts were reduced by PGPSt, although GSH expression was elevated. Apoptosis of hepatocytes is inhibited by PGPSt, which could be due to suppression of Bax and cas3, and overexpression of Bcl-2. Furthermore, western blot investigations demonstrated that PGPSt inhibited the synthesis of NF-*κ*B p65, p-P38, and TRL4 proteins. In mice, PGPSt protected them against LPS/D-GalN-induced ALI. In conclusion, the findings showed that PGPSt is an efficient therapeutic medication that increases the production of antiapoptotic proteins, lowers inflammatory mediators, and reduces OS, and might be applied to treat ALI in the future [88]. In LPS-induced mice, treatment with apigenin and myricetin (myricetin and apigenin, 100 and 200 mg/kg, PO) increased total protein levels, decreased serum levels of ALP, AST, ALT, CRP, *γ*-GT, direct and total bilirubin rates, MDA, NOx, and liver MPO activity. The liver structure damaged by LPS treatment was significantly restored by apigenin and myricetin treatment. Pre-treatment with myricetin and apigenin decreased liver damage indicators, OS, and inflammatory events, suggesting that these flavonoids may have hepatoprotective properties in ALI [89].

Pretreatment with *Lepidium sativum* (LS) (50 mg/kg) significantly reduced serum ALT and AST rates in the LPS-induced mice. The results showed a rise in TNF-*α* protein rates in the liver of mice administered with LS. The inflammatory mediator generation of IL-6, IL-10, IL-4, TGF-*β*, and IFN-*γ* was also reduced after administration with LS. Treatment with LPS may cause abnormalities such as sinusoid congestion, neutrophil infiltrations, necrosis, and hepatocellular decay, whereas LS therapy reduced these effects. These data suggest that LS has considerable hepatoprotective properties in ALI induced by LPS in mice [90].

Treatment with *Galaxaura oblongata* (*G. oblongata*) (200 mg/kg, IP) extract in LPS-induced mice notably reduced serum cytokines, such as LPO, NF-*κ*B, and MPO, and ameliorated apoptosis of the liver by suppressing the protein tyrosine kinase (PTK), which could be because of antioxidative properties of G. oblongata extract. The MDA level was suppressed in G. oblongata therapy. The marine extract of G. oblongata was shown to protect mice from acute liver damage in the research [91].

**Figure 3:**
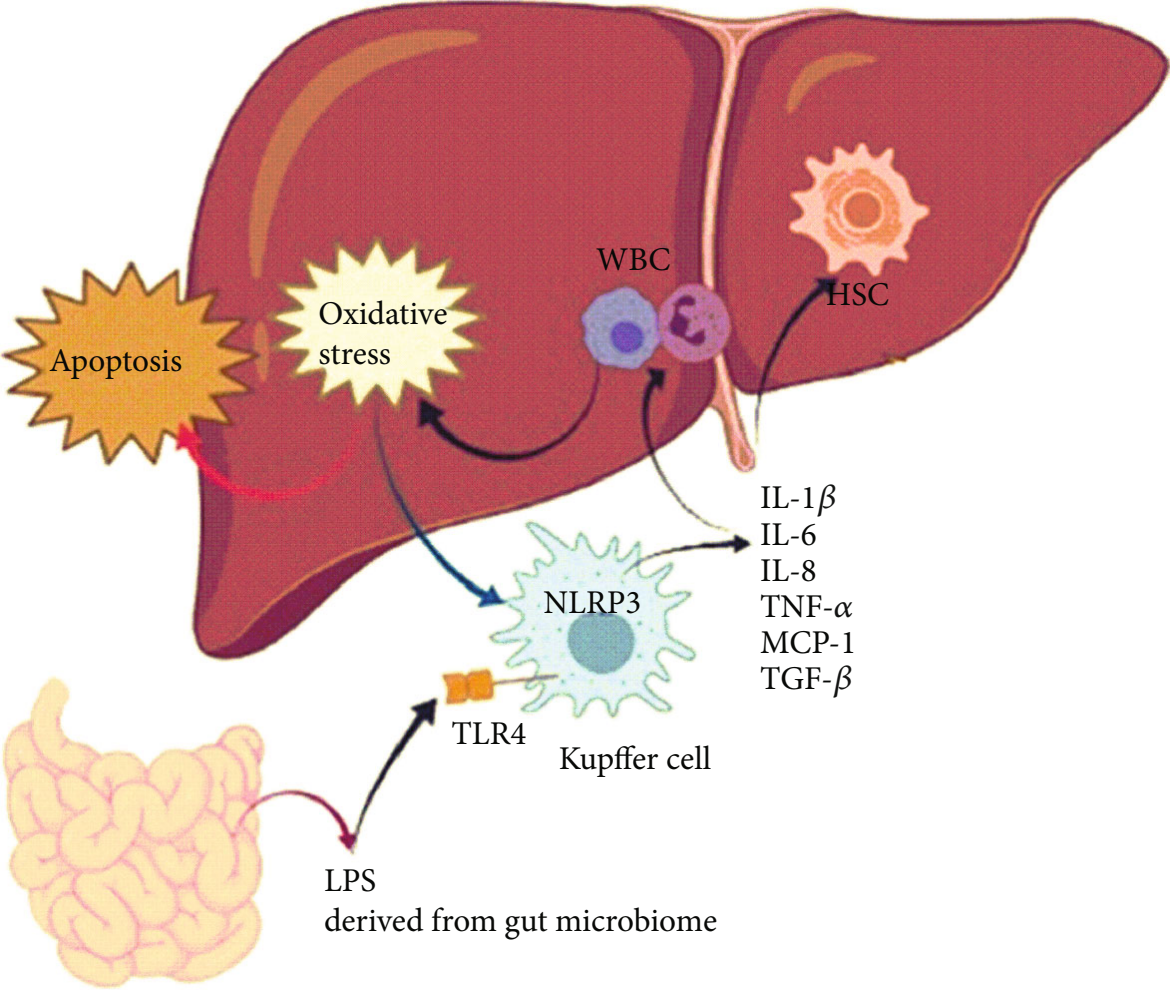
Lipopolysaccharide- (LPS-) induced hepatotoxicity. Toll-like receptor 4 (TLR4). Transforming growth factor beta (TGF-*β*). Monocyte chemo-attractant protein-1 (MCP-1).

Treatment with aminoguanidine (AG) (50, 100, and 150 mg/kg, IP) reduced LPS-enhanced liver toxicity by lowering MDA, NO, and IL-6 rates while enhancing total thiols, CAT, and SOD activity. Regarding the preventive role of AG seen in this investigation, AG appears that could reduce the elevated NO rates caused by iNOS activation in LPS-enhanced hepatotoxicity. When compared to the LPS group, AG injection improved serum albumin. In the present article, AG reduced NO, MDA, and OS metabolite levels while boosting total thiol group levels, as well as SOD and CAT activity in liver tissue in AG-treated groups [92]. The effects of medicinal herbs and natural products on LPS-Induced hepatotoxicity are shown in Table 3 and Figure 3.

## 6. Effects of Natural Products on LPS-Induced Immunomodulation

The *in vitro* anti-inflammatory activity of the ethyl acetate fraction of *Agarum cribrosum* in LPS-stimulated macrophage cell line (RAW264.7 cells) reduced the NO production. The expression of IL-1, IL-6, COX-2, and TNF-*α* was significantly suppressed by *A. cribrosum* fractions (5, 10, and 20 *μ*g/mL). The anti-inflammatory activity was also confirmed by studying its effects on proinflammatory signaling pathways. The suppression of NF-*κ*B p-65 inhibited the activation of NF-*κ*B and MAPKs. As a result, trifuhalol A (a phlorotannin extracted from *Agarum cribrosum*) can be used in the prevention or treatment of inflammation [93].

Recently, LPS-induced RAW macrophages were used to test the efficacy of tamarind extract. The effect of tamarind extract on the generation of NO in RAW macrophages was investigated. LPS-unstimulated macrophages (control) produced the least amount of NO, whereas the LPS-stimulated group produced more. The generation of NO was reduced in the presence of tamarind (3.175 to 150 *μ*g/mL) extract at the dosages examined. LPS-stimulated RAW macrophages significantly increased iNOS gene expression. iNOS levels were considerably reduced in the presence of tamarind extract at different dosages, compared to LPS treatment alone. The aqueous fruit pulp extract's inhibitory action on NO generation and iNOS gene expression make it an excellent anti-inflammatory treatment in situations when NO production is in excess [94]. Essential oils of *Amomum aromaticum* (0.1 *μ*g/ml to 100 *μ*g/ml) decreased NO production in LPS-induced RAW264.7 Cells (*In vitro*) by preventing expressions of COX-2 and iNOS [95].

Crocetin improved cell cytotoxicity and suppressed MCP-1 and the expression of IL-8 through blocking nuclear factor-kappaB p65 (NF-*κ*B p65) activity after LPS-induced inflammatory responses in Human umbilical vein endothelial cells (HUVEC) cells. Furthermore, crocetin inhibited immune cell infiltration, adhesion molecules expression, and ameliorated dysfunction endothelium [96]. These results indicated that crocetin showed protective effects on induced vascular injury by reduction of inflammation.

It was shown that quercetin as an immunoregulatory agent dependently impaired the high production of chemokines and cytokines in LPS-stimulated dendritic cells (DCs). Generation of cytokines such as IL-1 *β*, TNF-*α*, IL-1 *α*, IL-6, IL-10, IL-12 p70, and chemokines such as MCP-1, macrophage inflammatory protein (MIP)-1 a, and MIP-1 b significantly decreased by quercetin treatment (6.25, 12.5, 50, and 100 *μ*g/ml) in LPS-stimulated DCs. Quercetin also significantly suppressed the increased expression of CD40, CD80, and CD86 in the DCs [97].

The effect of *Portulaca oleracea* ethanol extract (50-200 *μ*g/ml) against LPS-induced inflammation in RAW 264.7 cells was examined. Results of this study showed this extract could inhibit the production of NO, IL-1*β*, TNF-*α*, and IL-6 in the cells. *P. oleracea* extracts inhibited the phosphorylation of ERK1/2, JNK, and activation of NF-*κ*B in the cells [98]. Treatment with 250 *μ*g/ml of a polysaccharide fraction purified from *P. oleracea* (POL-P3b) upregulated the expression of CD80, CD83, and CD86 in DCs. Also, the production of TNF-*α*, IL-12, and to a lesser extent IL-10 were upregulated by POL-P3b. In addition, POL-P3b increased the expression of TLR-4 in DCs [99].

Administration of thymoquinone (10 *μ*M) on LPS-activated mast cells restored LPS-induced changes at mRNA and protein levels of IL-5 and IL-13 but did not affect IL-10 production. Thymoquinone also inhibited GATA transcription factor binding at the IL-5 promoter induced by LPS stimulation [100]. Treatment with thymoquinone (1-20 *μ*M) also significantly inhibited LPS-induced IL-12, IL-10, and TNF-*α* release from DCs and suppressed LPS-induced phosphorylation of Protein kinase B (PKB)/Akt and ERK1/2 but induced caspase-3 and caspase-8 activity in DCs [101].

The effects of linalool on LPS-induced inflammation in both *in vivo* and *in vitro* were evaluated. Linalool (25 mg/kg, IP) inhibited the phosphorylation of inhibitor alpha gene (IjBa) protein, JNK, p38, and extracellular signal-regulated kinase in LPS-stimulated RAW 264.7 cells. Linalool also significantly reduced the production of inflammatory cytokines in the BALF of mice with LPS-induced lung injury and attenuated histopathological changes of lung tissues compared to the LPS group [102].

**Figure 4:**
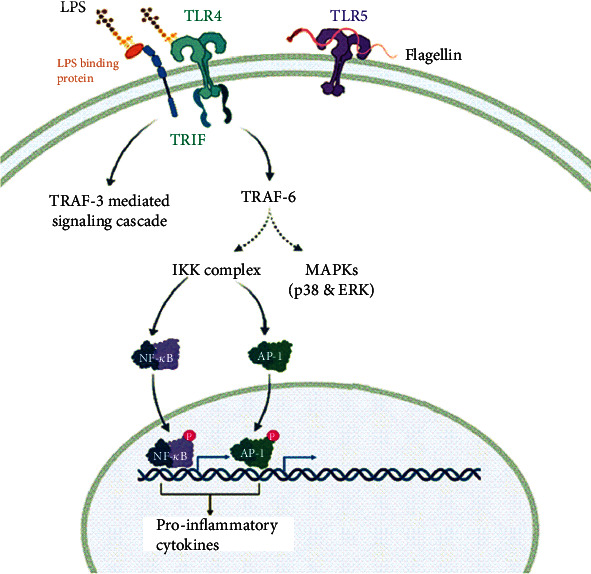
Lipopolysaccharide- (LPS-) induced immunomodulation. Lipopolysaccharides (LPS). Nuclear transcription factor-kappa B (NF*κ*B). Toll-like receptor 4 (TLR4). The mitogen activated protein kinases (MAPKs). Janus kinase-signal transducers and activators of transcription (JAK-STAT).

The extract of *Curcuma longa* significantly increased the level of NO, IL-2, IL-6, IL-12, IL-10, IFN-*γ* TNF-*α*, and MCP-1 in non-LPS stimulated mouse splenocytes and mouse macrophages. This extract also inhibited the production of PGE2 and IL-12 in LPS-stimulated cells [103]. The effects of medicinal herbs and natural products on LPS-Induced immunomodulation are shown in Table 4 and Figure 4.

## 7. Conclusion

*In vitro* and *in vivo* studies reported that the possible potential therapeutic effects of herbal medicine and natural products on LPS-induced toxicity in different organs. The preventive or prophylactic effects of natural products on LPS-induced toxicity may be due to their anti-inflammatory, antioxidant, and immunomodulatory properties. Therefore, the results of the current review study suggested the therapeutic effect of natural products on inflammation and oxidative stress pathway. However, these findings provide information about natural products for the prevention and treatment of LPS-induced toxicity, but the scientific validation of these compounds requires clinical trials and evidence on animal models for replacing modern commercial medicine.

## Figures and Tables

**Table 1:** Effects of natural products on LPS-induced neuroinflammation.

Natural product	Doses and administration	Study design	Effects	Ref.
Crocin	20 *μ*M	Rat brain microglial cells	↓ NO production ↓ TNF-*α* production ↓ IL-1*β* production ↓ NF-*κ*B activity	[26]
Crocetin	40 *μ*M			
*Tripterygium wilfordii*	—	Neuron-glia cells	↓ Level TNF*α* and NO	[29]
Myricetin (extracted from red wine)	12.5, 25, 50 *μ*M	BV-2 murine microglia cell line	↓ Levels of the TNF-*α*, IL-1*β* and IL-6 mRNA ↓ Levels of the TNF-*α*, IL-1*β* and IL-6 proteins Suppression of MAPK and NF-*κ*B pathways	[28]
Biochanin	1.25, 2.5, 5 *μ*M	Rat primary microglia	↓ Levels of NO, TNF-a, ROS, and IL-1b	[31]
Tripchlorolide (extract of *Tripterygium wilfordii*)	1.25–10 nM	Primary cortical neurons and neuro-2A cells	Inhibition of iNOS and COX-2 ↓ Production of TNF-a, IL-1b, NO, PGE2, superoxide anion	[30]
Licochalcone A (a flavonoid isolated from licorice roots)	0.625, 1.25, and 2.5 *μ*g/ml	BV-2 cells	Inhibiting phosphorylation of NF-KB p65 and ERK1/2 ↓ Production of NO and PGE2 ↓ Expression of iNOS and COX-2. ↓ Production of TNF-*α*, IL-1*β*, IL-6 Prevention of degeneration of dopaminergic neurons	[32]
Silymarin (a milk thistle polyphenolic flavonoid)	10, 20, 40, 60, 80, and 100 *μ*M	Mixed mesencephalic neuron–glia cultures	↓ Levels of TNF-*α* and NO ↓ Expression levels of iNOS protein and mRNA Inhibition of NF-*κ*B ↓ Superoxide generation	[33]
Carvacrol	25, 50, and 100 mg/kg, IP	Rats	↓ NO, IL-6 and MDA level ↑ CAT, SOD activity ↑ Total thiol content	[34]
Carvacrol	25, 50, and 100 mg/kg, IP	Rats	↓ IL-1*β*, TNF-*α*, TLR4, and COX-2 expression ↑ BDNF expression	[35]
Gypenosides	25, 50, and 100 mg/kg, ICV	Rats	↓ NF-*κ*B, IL-6, IL-1*β* level ↓ TLR4 and BDNF expression	[36]
Aqueous *Clinacanthus nutans* extract	500 and 1000 mg/kg, oral gavage	Rats	Improvment in metabolism of isoleucine, leucine, valine and pyruvate Regulation of metabolic pathway glycolysis, gluconeogenesis, and TCA cycle	[37]
*Euphausia superba* oil	80 mg/kg/day, feeding (rodent chow supplemented with 5 wt % of *Euphausia superba* oil)	Mice	↓ Expression of COX-2 and iNOS ↓ Level of MDA Suppress of translocation of p50 and p65 into the nuclei ↓ APP expression ↓ BACE1 expression	[38]
*Nannochloropsis oceanica*	50, 100 mg/kg, Administration of ethanol extract in drinking water	Mice	↓ Expression of COX-2 and iNOS ↓ Level of MDA Suppress of translocation of p50 and p65 into the nuclei ↓ APP expression ↓ BACE1 expression	[39]
Forsythoside B	1 and 2.5 *μ*M	BV-2 cells and HT22 cells	Inhibition of JNK, WDFY1/TLR3, and NF-*κ*B pathway	[40]
Forsythoside B	400 mg/kg, intragastrically	C57 mice	Inhibition of JNK, WDFY1/TLR3, and NF-*κ*B pathway	[40]
6-Oangeloylplenolin (isolated from *Centipeda minima*)	5−20 mg·kg, IP	Mice	↓ Expression levels of TNF-*α* and IL-1*β* ↓ Protein levels of iNOS, COX-2, phospho-NF-*κ*B p65, NF-*κ*B p65, phospho-I*κ*B-*α*, and I*κ*B-*α*	[41]
6-Oangeloylplenolin (isolated from *Centipeda minima*)	0.5−4 *μ*M	BV2 and primary microglial cells	↓ Protein levels of NF-*κ*B p65, phospho-NF-*κ*B p65, I*κ*B-*α*, and phospho-I*κ*B-*α* ↓ Expression of iNOS and COX-2 ↓ Concentration of NO and PGE2 ↓ Expression of NOX-2 and NOX-4	[41]
Myricetin (extracted from red wine)	2.5, 5, 10 mg/kg, IP	Rats	↓ Number of Iba-1-positive cells ↓ Levels of the Iba-1 protein ↓ Expression of TNF-*α*, IL-1*β*, and IL-6	[28]
Biochanin	12.5, 25, 50 mg/kg, IP	Rats	↓ Secretion IL-1b, IL-6, and TNF-*α* Suppress of ERK, JNK, p38 phosphorylation Block of MAPK signaling pathway	[31]
Licochalcone A (a flavonoid isolated from licorice roots)	1.25, 2.5, and 5 mg/kg, IP	Rats	↓ Expression of iNOS, COX-2, TNF-*α*, IL-6, and IL-1 *β* in the substantia nigra pars compacta region	[32]
Tenuigenin (an active component of *Polygala tenuifolia)*	300 mg/kg	Rats	Prevention of degeneration of dopaminergic neurons. ↓ TNF-*α* and IL-1b levels in the substantia nigra pars compacta and blood	[42]
Hydroxysafflor yellow A (a component extracted from Flos *Carthami tinctorii)*	50 and 100 *μ*M	Cortical neuron and microglial cells	↓ TLR4 expression in the microglia ↓ The numbers of apoptotic neurons ↓ Levels of the cleaved caspase-3 protein in the neurons ↓ Expression, phosphorylation, and nuclear translocation of MyD88, NF-*κ*B p65 in the microglia ↓ Levels of the MyD88 protein in the neurons ↓ p-ERK1/2, p-JNK, and p-p38 expression in the microglia ↓ TNF-a, IL-1b, and NO levels in the microglia ↑ Expression of BDNF in the microglia	[43]

ICAM-1: intercellular adhesion molecule-1; VCAM-1: vascular cell adhesion molecule-1; MPO: myeloperoxidase; CAT: catalase; SOD: superoxide dismutase; ROS: reactive oxygen species; TLR4: Toll-like Receptor 4; MDA: malondialdehyde; COX2: cyclooxygenase-2; GSH: glutathione; MCP-1: monocyte chemoattractant protein-1; W/D: wet/dry; NF-*κ*B: nuclear factor-kappa B; lactate dehydrogenase; KC: keratinocyte-derived chemokine; 4-HNE: 4-hydroxynonena; C+R+I: chlorogenic acid+rosmarinic acid+isofraxidin; PMNs: polymorphonuclear leukocytes; AECs: alveolar epithelial cells; iNOS: inducible nitric oxide synthase; PMVECs: pulmonary microvascular endothelial cells; MAPKs: mitogen-activated protein kinases; JNK: Jc-Jun-NH2 terminal kinase; GRP78: glucose-regulated protein 78; p-IRE1*α*: phosphorylated inositol-requiring enzyme 1*α*; TXNIP: thioredoxin interaction protein; IRE1*α*: inositol-requiring enzyme 1*α*; GSH-Px: glutathione peroxidase; ICV: intracerebroventricularly; IP: intraperitoneal injection. Per os (PO) or Oral administration; IV: intravenous; IG: intragastric.

**Table 2:** Effects of natural products on LPS-induced lung inflammation.

Natural product	Doses	Study design	Effects	Ref.
Procyanidin B2	5, 10, 20 mM	Alveolar epithelial cells (AECs)	↓ Annexin V ↓ NLRP3, ↓ NF-kB ↓ ROS ↓ TNF-*α*, ↓ IL-1*β* ↓ Bax, ↓ caspase-3 ↑ Bcl-2	[44]
Lonicerin	From 0 to160 *μ*M	BEAS-2B cell line	↓ IL-6, ↓ TNF-*α*, ↓ IL1*β* ↓ TLR4, ↓ MD2, ↓ p-NF-*κ*B ↓ Caspase-3, ↓ PARP	[45]
*Spiraea prunifolia*	50 , 100 mg/kg, PO.	NCI-H292 cells in mouse model	↓ Lipid Peroxidation ↓ IL-1*β*, ↓ IL-6, ↓ TNF-*α* ↓ MAPKs, ↓ NF-*κ*B ↓ OS, ↓ ROS, ↓DPPH radicals, ↓ GSH ↑ Nrf2, ↑ HO-1, ↑ NQO1	[46]
S-allylmercaptocysteine	10, 30, and 60 mg/kg, IG.	Male BALB/c mice	↓ Macrophages, ↓ Neutrophils ↓ TNF-*α*, ↓ IL-1*β*, ↓ IL-6 ↓OS, ↓ MPO ↓ NF-*κ*B activation ↓ MDA, ↓ iNOS, ↓ COX2 ↓ Nrf2 pathway ↑ SOD, ↑ GSH	[47]
Robustaflavone-4′-dimethyl ether	50, 100, and 200 mg/kg, IG.	ICR mice	↓ FLT3 ↓ AKT, ↓MAPK ↓ Neutrophils, ↓ MPO ↓ IL-6, ↓ TNF-*α* ↓ P-selectin, ↓ ICAM-1	[48]
Eriodictyol	20, 40, and 80 mg/kg, PO.	Male BALB/c mice	↓ Macrophages, ↓ Neutrophils ↓ MPO, ↓ MDA ↓ COX-2, ↓ NLRP3, ↓ NF-*κ*B ↓ PGE2, ↓ IL-6, ↓ TNF-*α*, ↓ IL-1*β* ↑ SOD, ↑ W/D ratio	[49]
*Trichilia martiana* C. DC.	5, 10, 20, and 40 *μ*g/ml, PO.	Male C57BL/6N mice	↓ Macrophages ↓ TNF-*α* ↓ I*κ*B, ↓ ERK, ↓ JNK ↑ HO-1	[51]
Pequi (*Caryocar brasiliense* Cambess)	20 mg/kg, PO.	Male ALI mice	↓ Leukocytes, ↓ Neutrophils ↓ TNF-*α*, ↓ IL-1, ↓ IL-6 ↓ MCP-1, ↓ KC ↓ MPO, ↓ MDA	[52]
*Glycyrrhiza glabra*	200, 300 mg/kg, PO.	Female mice	↓ Cell migration ↓ W/D ↓ Lung edema ↓ Protein content ↓ TNF-*α* , IL-1*β*, and IL-6 ↑ SOD activity	[53]
*Thalictrum minus* L.	10, 20, 40 mg/kg, PO.	C57 male mice	↓ W/D weight ratio ↓ Total protein ↓ NO, ↓ TNF-*α*, ↓ IL-1*β* ↓ MAPKs, ↓ p38-NLRP3, ↓ Caspase-1 ↓ COX2 ↓ Bax, ↓ LC3II, ↑ Phosphorylation of AMPK ↑ Bcl-2, ↑ Nrf2 ↑ SOD	[54]
Tovophyllin A	50 or 100 mg/kg, PO.	Male BALB/c albino mice	↓ LDH activity, ↓ Total protein, ↓ W/D ratio ↓ Inflammatory cells ↓ Lung lesions ↓ TNF-*α*, ↓ IL-6, ↓ IL-1*β* ↑ GSH, ↑ SOD	[55]
Peiminine	1, 3, 5 mg/kg, IP.	Male BALB/c mice	↓ W/D ratio ↓ TNF-*α*, ↓ IL-1*β*, ↓ IL-6 ↓ Neutrophils, ↓ Macrophages. ↓ MPO ↓ NF-*κ*B, ↓ PI3K, ↓ AKT	[56]
*Euphorbia cuneata*	25 and 50 mg/kg, PO.	Male Balb/c albino mice	↓ W/D ratio ↓ Total protein ↓ TNF-*α*, ↓ IL-8, ↓ IL-4 ↓ LDH ↓ Neutrophils, ↓ Macrophage, ↓ Lymphocyte ↓ MDA, ↓ 4-HNE ↓ NF-*κ*B , ↓ COX ↓ Lipid peroxidation ↑ CAT, ↑ SOD, ↑ GSH	[50]
*Nigella sativa*	100, 200, 400 mg/kg, IP.	Male Wistar rats	↓ Eosinophils, ↓ Neutrophils, ↓ Basophils, ↓ Monocytes ↓ INF*γ*, ↓ TGF-*β*1, ↓ PGE2, ↓ NO, ↓ IL-6 ↓ MDA ↑ IL-4, ↑ CAT, ↑ SOD ↑ Thiol level	[57]
Narciclasine	2 mg/kg, IP.	Neonatal rats	↓ TNF-*α*, ↓ IL-6, ↓ IL-1*β*, ↓ MCP-1 ↓ ICAM-1, ↓ VCAM-1 ↓ ROS, ↓ Cell apoptosis ↓ TLR4, ↓ MyD88, ↓ COX2 ↓ p-I*κ*B*β*	[58]
Chrysin	3 mg/kg, IT.	Male ICR mice	↓ MPO ↓ IL-1*β*, ↓ IL-6, ↓ TNF-*α* ↓ GRP78, ↓ p-IRE1*α* ↓ IRE1*α*, ↓ TXNIP, ↓ NLRP3 ↑ SOD, ↑ GSH-Px	[59]
Ergosterone	15 and 30 mg/kg, PO.	ALI model mice	↓ W/D ratio ↓ Pulmonary edema ↓ TNF-*α*, ↓ IL-1, ↓ IL-6, ↓ NO ↓ P-selectin, ↓ ICAM-1 ↓ MDA, ↓ NLRP3 ↑ SOD	[60]
Ferulic acid	25, 50, 100 mg/kg, IG.	Female BALB/c mice	↓ Cells infiltration ↓ Neutrophils, ↓ Macrophages ↓ Lung edema ↓ W/D ratio ↓ MPO, ↓ MCP-1 ↓ TNF-*α*, ↓ IL-1*β*, ↓ IL-6 ↓ TLR4, ↓ NF-*κ*B	[61]
Fucoxanthin	10 mg/kg, IV.	ALI model mice	↓ COX-2, ↓ iNOS, ↓ IL-10, ↓ IL-6 ↓ TNF-*α*, ↓ IL-1*β* ↓ NF-*κ*B ↓ TLR4, ↓ MyD88 ↑ Nrf2 p	[62]
Lonicerin	10, 20, and 30 mg/kg, IP.	Male C57BL/6 mice	↓ W/D weight ratio ↓ Total protein ↓ PMNs, ↓ Macrophages ↓ IL-6, ↓ TNF-*α*, ↓ IL-1*β* ↓ TLR4, ↓ MD2, ↓ p-NF-*κ*B ↓ Bax, ↓ Bcl-2 ↓ Caspase-3, ↓ PARP	[45]
Dehydrodieugenol B	20, 30, and 60 mg/kg/weight	Male BALB/C mice	↓ Lung edema, ↓ Inflammatory cells, ↓ IL-6, ↓ IL-1 *β* ↓ iNOS, ↓ MMP-9, ↓ TIMP-1 ↓ JNK	[63]
chlorogenic acid+rosmarinic acid+isofraxidin	5 to 50 mg/kg, IP.	Female and male BALB/c mice	↓ Inflammatory cell ↓ TNF-*α*, ↓ iNOS, ↓ COX-2, ↓ IL-6 ↓ W/D ratio ↓ MPO, ↓ NF*κ*B p65 ↓ OS, ↓ NO ↑ SOD, ↑ HO-1	[64]
Salviplenoid A	10, 20, and 40 mg/kg, IP.	Male BALB/c mice	↓ TNF-*α*, ↓ IL-6, ↓ IL-8, ↓ IFN-*γ* ↓ COX-2 ↓ Eosinophils ↑ Nrf2, ↑ HO-1, ↑ GCLC	[65]
Thymol	20-80 mg/kg, IP.	Male BALB/c mice	↓ Histopathological changes, ↓ IL-6, ↓ TNF-*α*, ↓ IL-1*β* ↓ MPO ↓ MDA ↓ NF-*κ*B activation	[66]
Carvacrol	20, 40, or 80 mg/kg, IP.	Male BALB/c mice	↓ Inflammatory cell ↓ TNF-*α*, ↓ IL-6 ↓ W/D ratio ↓ IL-1*β*	[67]
Hordenine	10 mg/kg	ALI mouse model	↓ Inflammatory cell ↓ TNF-*α*, ↓ iNOS, ↓ COX-2, ↓ IL-6 ↓ IL-1*β* ↓ NF-*κ*B	[68]
Munronoid I	10 mg/kg, IV.	ALI mouse model	↓ Inflammatory cell ↓ TNF-*α*, ↓ IL-6 ↓ IL-1*β* ↓ Scored of lung tissue damage	[69]

ICAM-1: intercellular adhesion molecule-1; VCAM-1: vascular cell adhesion molecule-1; MPO: myeloperoxidase; CAT: catalase; SOD: superoxide dismutase; ROS: reactive oxygen species; TLR4: Toll-like Receptor 4; MDA: malondialdehyde; COX2: cyclooxygenase-2; GSH: glutathione; MCP-1: monocyte chemoattractant protein-1; W/D: wet/dry; NF-*κ*B: nuclear factor-kappa B; LDH: lactate dehydrogenase; KC: keratinocyte-derived chemokine; 4-HNE: 4-hydroxynonena; C+R+I: chlorogenic acid+rosmarinic acid+isofraxidin; PMNs: polymorphonuclear leukocytes; AECs: alveolar epithelial cells; iNOS: inducible nitric oxide synthase; PMVECs: pulmonary microvascular endothelial cells; MAPKs: mitogen-activated protein kinases; JNK: Jc-Jun-NH2 terminal kinase; GRP78: glucose-regulated protein 78, p-IRE1*α*: phosphorylated inositol-requiring enzyme 1*α*; TXNIP: thioredoxin interaction protein; *α*IRE1*α*: inositol-requiring enzyme 1; GSH-Px: glutathione peroxidase.

**Table 3:** Effects of natural products on LPS-induced hepatotoxicity.

Natural product	Doses	Study design	Effects	Ref.
Mangiferin	—	*In vitro,* liver-resident Kupffer cells	↓ TNF-*α*, ↓ NF-*κ*B, ↓ AP-1 ↓ TLR4, ↓ HO-1	[70]
Apigenin	2.5, 5, 10, and 20 *μ*M	*In vitro,* rat BRL hepatocytes	↓ MDA, ↓ TNF-*α* ↑ Nrf2, ↑ PPAR, ↑ I*κ*B-*α*	[71]
Limonin	10, 25, and 50 *μΜ*	*In vitro,* HepG2 cells	↓ ROS, ↓ NLRP3, ↓ cas-1 ↓ IL-1*β*	[72]
Ethanol extract of *Illicium henryi* (EEIH)	1.25, 2.5, and 5.0 mg/kg, IP.	Male BALB/c mice	↓ IL-1*β*, ↓ IL-6, ↓ TNF-*α*, ↓ COX-2 ↓ TLR4, ↓ NF-*κ*B, ↓ NO, ↓ iNOS ↓ ALT, ↓ SLT, ↓ MPO ↑ Nrf2, ↑ GSH, ↑ SOD	[73]
Kaempferol	2.5, 5, 10, 20, and 40 mg/kg, IV.	Male wild-type mice	↓ CHOP, ↓ Grp78, ↓ ER stress	[74]
Chicoric acid (CA)	50 mg/kg	Male C57BL/6 mice	↓ AST, ↓ ALT, ↓ ROS ↓ MAPKs, ↓ NF-*κ*B, ↓ NLRP3 ↓ Cas-1, ↓ ASC ↑ GSH, ↑ Nrf2, ↑ AMPK	[75]
Myricetin (Myr)	25, 50, or 100 mg/kg, IP.	Male C57BL/6 mice	↓ AST, ↓ ALT, ↓ OS ↓ IL-6, ↓ IL-1*β*, ↓ TNF-*α* ↓ Cas-3, ↓ Cas-9 ↓ TLR4, ↓ NF-*κ*B, ↓ MAPK ↑ Nrf2, ↑ HO-1, ↑ ACC	[76]
Limonin	50 and 100 mg/kg, PO.	C57BL/6 mice	↓ AST, ↓ ALT, ↓ LDH ↓ IL-1*β*, ↓ Pyroptosis ↑ GSH	[72]
Mangiferin (MF)	30, 100, or 150 mg/kg, PO.	BALB/c mice	↓ ALT, ↓ AST, ↓ TNF-*α* ↓ NF-*κ*B, ↓ TLR4 ↑ HO-1	[70]
Mungbean seed coat water extract (MSWE)	150 mg/kg, PO.	Male ICR mice	↓ iNOS, ↓ IL1b, ↓ TLR4 ↓ Emr1	[77]
Diosgenin	50 mg/kg, PO.	Male C57BL/6 mice	↓ AST, ↓ ALP, ↓ ALT ↓ IL-6, ↓ IL-1*β*, ↓ MDA, ↓ ROS, ↓ NF-*κ*B, ↓ TNF-*α*, ↓ TRL4, ↓ MPO, ↓ OS ↑ Nrf2, ↑ SOD	[78]
Shiitake mushroom-derived ELNs (S-ELNs)	1 × 10^10^/g, IP.	C57BL/6 J mice	↓ NLRP3, ↓ AST, ↓ ALT ↓ IL-18, ↓ IL-6, ↓ IL-1*β*	[79]
Ganoderma lucidum (G. lucidum)	5, 10, and 20 mg/kg	Female BALB/c mice	↓ MyD88, ↓ TLR4, ↓ NF-*κ*B, ↓ MAPK ↓ p38, ↓ JNK, ↓ ErK1/2 ↓ IL-6, ↓ TNF-*α*, ↓ AST, ↓ALT	[104]
S-allyl cysteine (SAC)	25 and 100 mg/kg, PO.	Male C57BL/6 mice	↓ AST, ↓ ALT, ↓ ALP ↓ ROS, ↓ MDA, ↓ OS ↓ FRAP, ↓ MDA, ↓ ROS ↓ cas-1, ↓ cox-2, ↓ TLR4, ↓ NF-*κ*B, ↓ TNF-*α*, ↓ NLRP3, ↓ IL-6, ↓ IL-1*β*, ↓ MPO, ↓neutrophils ↑cas-3	[80]
*Pulicaria petiolaris* (PP)	50 and 100 mg/kg, PO.	Male Swiss albino mice	↓ AST, ↓ ALP, ↓ ALT ↓ MDA, ↓ NF-*κ*B, ↓ IL-6, ↓ TNF-*α* ↓ LDH, ↓ CK-MB ↑ GSH, ↑ SOD	[81]
Apocynin	300 mg/kg, IP.	Male BALB/c mice	↓ ROS, ↓ TBARS ↓ TNF-*α*, ↓ OS, ↓ mortality ↓ AMPK, ↓ JNK	[82]
*Beta vulgaris* ethanol extract (BVEE)	200 and 400 mg/kg, PO.	Male Sprague–Dawley rats	↓ AST, ↓ ALT, ↓ *γ*-GTP ↓ CYP2E1, ↓ MAPK1, ↓ MAPK3 ↓ *α*-SMA, ↓ NF-*κ*B ↑ NO	[83]
Curcumin (CUR)	200 mg/kg suspended in 1% CMC	Male albino Wistar rats	↓ LDH, ↓ AST, ↓ ALT ↓ GSH, ↓ SOD, ↓ MDA, ↓ NO ↓ CRP, ↓ IL-6, ↓ TNF-*α*, ↓ TLR4 ↓ NF-*κ*B, ↓ JNK, ↓ p38, ↓ OS ↑ HO-1	[84]
Apilarnil (API)	0.2, 0.4, and 0.8 g/kg, PO.	Sprague-Dawley rats	↓ XOD, ↓ TCN1, ↓ MDA ↓ NF-*κ*B, ↓ TLR4, ↓ HMGB-1 ↓ iNOS, ↓ IL-6, ↓ IL-1*β*, ↓ TNF-*α* ↑ CAT, ↑ SOD	[85]
Heptamethoxyflavone (HMF)	500 mg/kg	Male albino adult rats	↓ ALT, ↓ ALP, ↓ AST ↓ MDA, ↓ Cas-3, ↓ Cas-9 ↓ Bax, ↓ Bcl-2, ↓ TNF-a ↓ IL-b, ↓ NF-jB	[86]
Esculetin	40 mg/kg, PO.	Male C57BL/6 mice	↓ IL-6, ↓ IL-1*β*, ↓ TNF-*α*, ↓ NF-*κ*B ↓ TLR4, ↓ neutrophils, ↓ OS ↓ AST, ↓ ALP, ↓ ALT	[87]
*Platycodon grandiflorus* polysaccharides (PGPSt)	100 and 200 mg/kg, IG.	SPF-BALB/c mice	↓ SOD, ↓ ALT, ↓ AST ↓ TNF-*α*, ↓ IL-1*β*, ↓ IL-6 ↓ Bax, ↓ Cas-3, ↓ MDA ↑ GSH	[88]
Apigenin and myricetin	Myricetin and apigenin 100 and 200 mg/kg, PO.	Male Balb/c mice	↓ ALP, ↓ AST, ↓ ALT ↓ CRP, ↓ *γ*-GT, ↓ PGE2 ↓ TNF-a, ↓ MDA, ↓ NO ↓ MPO, ↓ ↓ IL-6, ↓ IL-1 ↓ Bilirubin, ↓ COX-2, ↓ iNOS ↓ NF-*κ*B, ↓ IKK, ↓ OS ↑ Total protein	[89]
*Lepidium sativum* (LS)	50 mg/kg	Male mice	↓ ALT, ↓ AST, ↓ TNF-*α* ↓ IL-6, ↓ IL-10, ↓ IL-4 ↓ TGF-*β*, ↓ IFN-*γ*, ↓ Neutrophils	[90]
*Galaxaura oblongata* (*G. oblongata*)	200 mg/kg, PO.	BALB/C mice	↓ LPO, ↓ NF-*κ*B, ↓ MPO ↓ PTK, ↓ MDA	[91]
Aminoguanidine (AG)	50, 100, and 150 mg/kg, PO.	Wistar rats	↓ MDA, ↓ NO, ↓ IL-6 ↓ NO, ↓ OS ↑ Total thiols, ↑ CAT, ↑ SOD	[92]

TNF-*α*: tumor necrosis factor-*α*; Nrf-2: nuclear factor erythroid 2-related factor 2; PPAR: peroxisome proliferator–activated receptor *γ*; CAT: catalase; SOD: superoxide dismutase; NF-*κ*B: nuclear factor-*κ*B; TLR4: Toll-like receptor 4; NLRP3: NOD-like receptor protein 3; Grp78: glucose-regulated/binding immunoglobulin protein 78; CHOP: C/EBP-homologous protein; ER stress: reticulum stress; AST: aspartate aminotransferase; ALT: alanine aminotransferase; ALP: alkaline phosphatase; MAPKs: mitogen-activated protein kinases; AMPK: AMP-activated protein kinase; HO-1: heme oxygenase-1; ACC: acetyl-CoA carboxylase; CK-MB: creatine kinase-MB; LDH: lactate dehydrogenase; TBARS: thiobarbituric acid reactive substances; HMGB-1: high-mobility group box protein 1; TCN-1: testican 1; OS: oxidative stress; CAS: caspase; IKK: inhibitor of nuclear factor kappa-B kinase; COX-2: cyclooxygenase 2; PTK: protein tyrosine kinase; LPO: lipid per oxidation; ICV: intracerebroventricularly, IP: intraperitoneal injection. Per os (PO) or oral administration; IV: intravenous; IG: intragastric.

**Table 4:** Effects of natural products on LPS-induced immunomodulation.

Natural product	Doses and administration	Study design	Effects	Ref.
*Agarum cribrosum*	5, 10 and 20 *μ*g/mL	RAW264.7 cells	↓ NO production ↓ TNF-*α*, ↓ IL-6, ↓ IL-1*β*, ↓ COX2 ↓ NF-*κ*B p65, ↓ MAPKs	[93]
*Tamarindus indica*	3.175 to 150 *μ*g/mL	RAW 264.7 macrophages	↓ NO production ↓ iNOS	[94]
*Amomum* *Aromaticum*	From 0.1 *μ*g/ml to 100 *μ*g/ml	Murine macrophage RAW264.7 cells	↓ NO production ↓ TNF*α*, ↓ IL-1*β*, ↓IL-6 ↓ Cox-2, ↓ iNOS	[95]
Dehydrodieugenol B	10, 20, 30, and 60 *μ*g/mL	RAW 264.7 macrophages	↓ NO, ↓ IL-1*β*, ↓ IL-6	[63]
*Sarcandra glabra*	C+R+I: 0.03, 0.06, and 0.06 mmol	RAW 264.7 macrophages	↓ IL-6, ↓ TNF-*α* ↓ MAPK, ↓ NF-*κ*B ↓ I*κ*B, ↓ COX-2, ↓ iNOS ↓ P-p38, ↓ P-JNK, ↓ P-ERK	[64]
Salviplenoid A	—	RAW 264.7 murine macrophage	↓ NO, ↓ TNF-*α* ↓ NF-*κ*B	[65]
Munronoid I	12.5 *μ*M, 25 *μ*M, 50 *μ*M	Mouse peritoneal macrophages	↓ COX-2, ↓ iNOS, ↓ IL-1*β*, ↓ TNF-*α*	[69]
Crocetin (saffron)	—	HUVEC	↓ MCP-1 expression ↓ IL-8 expression ↓ NF-*κ*B p65 activity ↓ Immune cells infiltration ↓ Adhesion molecules expression	[96]
Quercetin	6.25, 12.5, 50, and 100 *μ*g/ml	Dendritic cells	↓ Generation of TNF-*α*, IL-1 *α*, IL-1 *β*, IL-6, IL-10, IL-12 p70 ↓ Generation of MCP-1, MIP-1*α*, and MIP-1 b Suppress of expression of CD40, CD80, and CD86	[97]
*Portulaca oleracea* ethanol extract	50, 100, and 200 *μ*g/ml	RAW 264.7 cells	↓ Production of NO, TNF-*α*, IL-1*β* and IL-6 ↓ Phosphorylation of ERK1/2, JNK ↓ Activation of NF-*κ*B	[98]
POL-P3b (a polysaccharide fraction purified from *P. oleracea*)	250 *μ*g/ml	Dendritic cells	↑ Expression of CD80, CD83, CD86, and MHC class II molecules ↑ Production of IL-12, TNF-*α*, and IL-10 ↑ Expression of TLR-4	[99]
Thymoquinone	10 *μ*M	Mast cells	↓ Levels of IL-5 and IL-13 mRNA ↓ Levels of IL-5 and IL-13 protein Inhibition of GATA transcription factor binding at the IL-5 promoter	[100]
Thymoquinone	1-20 *μ*M	Dendritic cells	↓ Release of IL-10, IL-12, and TNF-*α* Suppress of phosphorylation of PKB/Akt, and ERK1/2 Induction of caspase-3 and caspase-8 activity	[101]
Linalool		RAW 264.7 cells	Inhibition of phosphorylation of IkB*α* protein, p38, c-JNK, and ERK ↓ Level of IL-6, TNF-*α*	[102]
Linalool	25 mg/kg, IP.	*In vivo*, mice	↓ Level of IL-6, TNF-*α* in the BALF	[102]
Aqueous extract of *Curcuma longa*	0.5, 4, 20, 100, 500	Mouse splenocytes and mouse macrophages	↑ Level of NO, IL-12, IL-10, IL-6, IL-2, TNF-*α*, IFN-*γ*, and MCP-1 in non-LPS-stimulated cells ↓ Production of IL-12 and PGE2 in LPS-stimulated cells	[103]

MCP-1: monocyte chemoattractant protein-1; IL-8: interleukin-8; NF-*κ*B p65: nuclear factor-kappa B p65; HUVEC: human umbilical vein endothelial cell; NO: nitric oxide; TNF-*α*: tumor necrosis factor-*α*; IL-1*β*: interleukin-1*β*; MDA: malondialdehyde, CAT: catalase; SOD: superoxide dismutase; TLR4: Toll-like receptor 4; BDNF: brain-derived neurotrophic factor; TCA: tricarboxylic acid; ICV: intracerebroventricularly; IP: intraperitoneal injection; COX-2: cyclooxygenase-2; APP: amyloid protein precursor; BACE1: *β*-site APP cleaving enzyme; MMP-3: matrix metalloproteinase 3; JNK: C-Jun NH2-terminal kinase; PGE2: Prostaglandin E2; Iba-1: ionized calcium binding adaptor molecule-1; MAPK: mitogen-activated protein kinases; ERK: Ras-dependent extracellular signal-regulated kinase; ROS: reactive oxygen species; MyD 88: myeloid differentiation factor 88; MIP-1*α*: macrophage inflammatory protein-1*α*; MHC: major histocompatibility complex; PKB/Akt: protein kinase B/serine-threonine kinase; IkB*α* protein: ubiquitination-inducible multiprotein.

## Data Availability

Data used to support the findings of this review are included within the article.
